# Race is an independent predictor of survival in patients with soft tissue sarcoma of the extremities

**DOI:** 10.1186/s12885-018-4397-3

**Published:** 2018-04-27

**Authors:** Alexander L. Lazarides, Julia D. Visgauss, Daniel P. Nussbaum, Cindy L. Green, Dan G. Blazer, Brian E. Brigman, William C. Eward

**Affiliations:** 1 Department of Orthopedic Surgery, Duke University Medical Center, Box 3000, Durham, NC 27710 USA; 2 Department of General Surgery, Duke University Medical Center, Durham, NC USA

## Abstract

**Background:**

In the United States, race and socioeconomic status are well known predictors of adverse outcomes in several different cancers. Existing evidence suggests that race and socioeconomic status may impact survival in soft tissue sarcoma (STS). We investigated the National Cancer Database (NCDB), which contains several socioeconomic and medical variables and contains the largest sarcoma patient registry to date. Our goal was to determine the impact of race, ethnicity and socioeconomic status on patient survival in patients with soft tissue sarcoma of the extremities (STS-E).

**Methods:**

We retrospectively analyzed 14,067 STS-E patients in the NCDB from 1998 through 2012. Patients were stratified based on race, ethnicity and socioeconomic status. Univariate and multivariate analyses were used to correlate specific outcomes and survival measures with these factors. Then, long-term survival between groups was evaluated using the Kaplan-Meier (KM) method with comparisons based on the log-rank test. Multiple variables were analyzed between two groups.

**Results:**

Of the 14,067 patients analyzed, 84.9% were white, 11% were black and 4.1% were Asian. Black patients were significantly more likely (7.18% vs 5.65% vs 4.47%) than white or Asian patients to receive amputation (*p* = 0.027). Black patients were also less likely to have either an above-median education level or an above-median income level (*p* < 0.001). In addition, black patients were more likely to be uninsured (*p* < 0.001) and more likely to have a higher Charleson Comorbidity Score than white or Asian patients. Tumors were larger in size upon presentation in black patients than in white or Asian patients (*p* < 0.001). Black patients had significantly poorer overall survival than did white or Asian patients (*p* < 0.001) with a KM 5-year survival of 61.4% vs 66.9% and 69.9% respectively, and a 24% higher independent likelihood of dying in a multivariate analysis.

**Conclusion:**

This large database review reveals concerning trends in black patients with STS-E. These include larger tumors, poorer resources, a greater likelihood of amputation, and poorer survival than white and Asian patients. Future studies are warranted to help ensure adequate access to effective treatment for all patients.

## Background

In the United States, race and socioeconomic status have been shown to predict adverse outcomes in various carcinomas. [1–3] Unfortunately, despite improvements in outcomes since the 1970s [4], we continue to see significant disparities among racial and ethnic groups. [5] Indeed, it appears that black patients exhibit higher mortality rates compared to other racial groups, even when controlling for cancer biology and treatment characteristics. [5, 6]

Existing evidence suggests that race may impact survival in soft tissue sarcoma (STS). [7, 8] Martinez et al. retrospectively examined 6706 patient primary soft tissue sarcoma of the extremities (STS-E). They found a number of striking differences in treatment and tumor characteristics amongst different races; specifically, they identified that black patients had decreased survival in a multivariate analysis [8]. A study by Alamanda et al. examined and similarly found that black patients had inferior overall outcomes compared to other races; however, they did not find that black race was an independent predictor of outcome when controlling for confounding factors [7]. Both of these studies utilized the Surveillance, Epidemiology, and End Results (SEER) database, which has limited outcomes data and does not capture all cancer diagnoses. The findings were conflicted as to whether black race is independently associated with poorer outcomes.

In this study, we investigated the National Cancer Database (NCDB), which contains several socioeconomic and medical variables and contains the largest sarcoma patient registry to date. Our goal was to determine the independent impact of race, ethnicity and socioeconomic status on treatment and outcomes in patients with soft tissue sarcoma of the extremities.

## Methods

The Institutional Review Board (IRB) of our institution approved this retrospective analysis of the NCDB of patients diagnosed with STS-E from 1998 through 2012. This database is a combined effort by the Commission on Cancer (CoC) of the American College of Surgeons and the American Cancer Society, and represents more than 70% of new cancer diagnoses every year from over 1500 accredited cancer programs in the U.S. [9] NCDB Participant User Files were accessed for patients treated at NCDB participating institutions; utilizing the International Classification of Diseases for Oncology, 3rd Edition (ICD-O-3) topography codes C47.1 (peripheral nerves of the upper limb and shoulder), C47.2 (peripheral nerves of the lower limb and hip), C49.1 (soft tissues of the upper limb and shoulder), and C49.2 (soft tissues of the lower limb and hip), we included only patients with extremity tumor location.

Patients were included who had primary, invasive STS-E. Race was divided into three groups based on Caucasian, black and Asian coding. Hispanic ethnicity is included as a separate category in the NCDB and was not specifically examined for the purposes of this study.

We compared 1) patients characteristic variables 2) tumor characteristics variables 3) treatment variables and 4) survival/ outcome variables. The specific patient variables included were as follows: age, sex, race, Charlson Comorbidity Score (CCS), income, education, facility type, distance to facility, insurance status; extremity location, tumor size, tumor histology, grade and TNM stage; type of surgery, adjuvant therapy utilization, surgical margins; and 30-day mortality rate, 30-day re-admission rate, overall survival.

We identified 14,067 STS-E patients in the NCDB from 1998 through 2012 eligible for retrospective analysis. Racial and socioeconomic differences with regards to tumor and treatment characteristics and patient outcomes were analyzed. Baseline characteristics and outcomes between groups were compared using Pearson’s chi-square test for categorical variables and analysis of variance (ANOVA) for continuous variables. Multivariable logistic regression was used to predict racial and socioeconomic characteristics that were associated with worse outcomes; all patient, tumor and treatment variables were included in this model. The long-term survival between the groups was evaluated using the Kaplan-Meier (KM) method with comparisons based on the log-rank test. *P*-values < 0.05 indicate statistical significance. All statistical analyses were performed using SAS (SAS Institute Inc., Cary, North Carolina).

## Results

Patient characteristics are summarized in Table 1. Of the 14,067 patients analyzed, 84.9% were white, 11% were black and 4.1% were Asian. White patients tended to be older at diagnosis than black or Asian patients (mean age 59.4 years, 54.1 years, and 55.2 years respectively, *p* < 0.001). Black patients were less likely to have either an above-median education level or an above-median income level (*p* < 0.001). In addition, black patients were more likely to be uninsured (p < 0.001) and more likely to have a higher Charleson Comorbidity Score than white or Asian patients.

**Table 1:** Baseline patient characteristics stratified by race

Variable	Overall (*n* = 13,148)	White (*n* = 11,162)	Black (*n* = 1449)	Asian (*n* = 537)	*p*-value
Age	58.7	59.4	54.1	55.2	< 0.001
Female	6077 (46.2)	5055 (45.3)	771 (53.2)	251 (46.7)	< 0.001
Charleson Comorbidity Score					< 0.001
0	10,986 (83.6)	9387 (84.1)	1147 (79.2)	452 (84.2)	
1	1769 (13.4)	1461 (13.1)	244 (16.8)	64 (11.9)	
>/= 2	393 (3)	314 (2.8)	58 (4)	21 (3.9)	
Education above median	7749 (58.9)	6973 (62.4)	481 (33.2)	296 (55.1)	< 0.001
Income above median	7926 (60.3)	6988 (62.6)	543 (37.5)	395 (73.6)	< 0.001
Distance Crowfly (mi)	48.7	51.4	29.9	42.6	*p* < 0.001
Uninsured	609 (4.6)	435 (3.9)	129 (8.9)	45 (8.4)	*p* < 0.001
Treatment facility					*p* < 0.001
Academic	7720 (58.7)	6454 (57.8)	914 (63.1)	352 (65.6)	
Non- Academic	5428 (41.3)	4708 (42.2)	535 (36.9)	185 (34.5)	

Tumor and treatment characteristics are summarized in Table 2. Tumors were larger in size upon presentation in black patients than in white or Asian patients (*p* < 0.001) and black patients were less likely to have tumors < 5 cm upon arrival (p < 0.001). Black patients were more likely to have well differentiated tumors than white or Asian patients, who were more likely to have higher grade tumors (*p* = 0.021). Black patients were significantly more likely (7.18% vs 5.65% vs 4.47%) than white or Asian patients to receive amputation (*p* = 0.027). Black patients were also more likely to not receive any surgery than white or Asian patients (p < 0.001). There was no difference in either time to surgery or time to radiation therapy (RT) from time of diagnosis (*p* = 0.7). There was no difference in the utilization of RT between races (*p* = 0.33). There were no statistically significant differences between margin negative post surgery rates between white, black or Asian patients (18.6%, 18.2% and 20.5% respectively).

**Table 2:** Patient treatment characteristics and outcomes stratified by race

Variable	Overall	White	Black	Asian	*p*-value
Tumor Size					*p* < 0.001
< 5 cm	3710 (28.2)	3261 (29.2)	319 (22)	130 (24.2)	
>/= 5 cm	9438 (71.8)	7901 (70.8)	1130 (78)	407 (75.8)	
Tumor Grade					*p* = 0.02
Well Differentiated	2803 (21.3)	2342 (21)	342 (23.6)	119 (22.2)	
Moderately Differentiated	2138 (16.3)	1818 (16.3)	226 (15.6)	94 (17.5)	
Poorly Differentiated	4811 (36.6)	4076 (36.5)	555 (38.3)	180 (33.5)	
Undifferentiated	3396 (25.8)	2926 (26.2)	326 (22.5)	144 (26.8)	
Chemo Usage	2568 (20)	2300 (20.8)	301 (21.3)	115 (21.9)	*p* = 0.70
RT Usage	7199 (55.3)	6127 (55.43)	771 (53.6)	301 (56.7)	*p* = 0.33
Margin Status					*p* = 0.51
Pos	2288 (18.7)	1949 (18.6)	237 (18.2)	102 (20.5)	
Neg	9965 (81.3)	8502 (81.4)	1068 (81.8)	395 (79.5)	
Surgical Procedure					*p* < 0.001
Amputation	759 (5.8)	631 (5.7)	104 (7.2)	24 (4.5)	
Radical Resection	7116 (54.1)	6114 (54.5)	727 (50.9)	265 (49.4)	
Local/ Partial Resection	4378 (33.3)	3706 (33.2)	464 (32)	208 (38.7)	
None	895 (6.8)	711 (6.4)	144 (9.9)	40 (7.5)	
Amputation (subanalysis)	759 (5.8)	631 (5.7)	104 (7.2)	24 (4.5)	*p* = 0.027
30 Day Mortality	50 (0.41)	46 (0.44)	3 (0.23)	1 (0.2)	*p* = 0.15

**Fig. 1:**
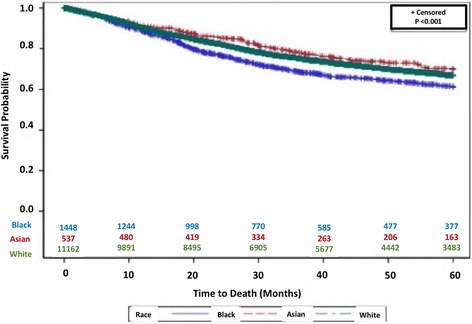
Kaplan Meier long-term survival for patients stratified by race

Outcomes are summarized in Table 2. There was no difference in 30 day survival between races (*p* = 0.15). Black patients had significantly poorer overall survival at 5 years follow up than did white or Asian patients (*p* < 0.001) with a KM 5-year survival of 61.4% vs 66.9% and 69.9% respectively (Fig. 1). There was no statistically significant difference in survival when stratifying by year of diagnosis (*p* = 0.37). In a multivariate analysis (Table 3), black race was found to be an independent predictor of adverse outcome, conferring a 24% (HR: 1.12–1.31) higher chance of death than white patients.

**Table 3:** Independent predictors of mortality in a multivariate analysis

Variable	HR	Lower 95%	Upper 95%	*p* value
PATIENT VARIABLES				
Age (per 5 year increase)	1.15	1.14	1.16	< 0.001
Male Sex	1.2	1.12	1.27	< 0.001
Race				
Black	1.24	1.12	1.36	< 0.001
Asian	0.9	0.76	1.07	0.226
Charlson Comorbidity				
1	1.24	1.14	1.34	< 0.001
2	1.86	1.62	2.15	< 0.001
Uninsured	1.39	1.19	1.63	< 0.001
% no HSD				
7–12.9%	1.14	1.05	1.24	0.002
13–20.9%	1.22	1.12	1.33	< 0.001
> 21%	1.29	1.17	1.42	< 0.001
TREATMENT VARIABLES				
Tumor Size (> 5 cm)	1.02	1.02	1.02	< 0.001
Grade				
Moderately Diff	2.16	1.85	2.51	< 0.001
Poorly Differentiated	4.43	3.91	5.01	< 0.001
Undifferentiated	4.58	4.04	5.2	< 0.001
Margin				
Macroscopic +	2.24	1.73	2.9	< 0.001
Microscopic +	1.13	1.02	1.26	0.018
No Surgery	4.3	3.94	4.7	< 0.001

## Discussion

Race has previously been demonstrated as a important variable in the outcomes of a number of cancer types. However, the role of race in STS has not been well delineated in prior literature, with conflicting evidence as to whether race is an independent driver of outcome. Using the NCDB, which captures over 70% of new cancer diagnoses every year, this study represents the largest study to date looking at the role of race in treatment and outcomes in STS-E. The goal of this retrospective cohort study was to examine the role of race in treatment decisions and outcomes of STS-E.

The U.S. Census currently reports that 30% of the U.S. populace identifies as a minority; by 2050, this number is projected to approach nearly 50%. [10] It stands to reason that racial and ethnic minority populations will represent an increasingly large constituent of the American healthcare system. However, over the past 20 years, little change is seen in cancer care for minorities. [11] As such, it is important to identify disparities in healthcare, specifically as they relate to race and socioeconomic status. According to the 2010 U.S. Census, white, black and Asian persons represented 72.1%, 12.6% and 4.8% of the population respectively at that time point. [12] Our study, which represents a cohort of patients from 1998 to 2012, had a near equivalent breakdown of minorities, though our patient cohort does have an overrepresentation of white patients (84.9%). It should be noted that the NCDB categorizes Hispanic patients separately from race as “Hispanic” or “Not Hispanic”. As such, we felt that our study adequately represented the U.S. population as a whole and served as a suitable model to investigate race.

Our model was consistent with accepted prognostic variables in soft tissue sarcomas of the extremities. In our multivariate analysis, the largest drivers of adverse outcomes were higher grade, positive margin status after resection, and greater number of patient comorbidities. Increasing tumor size and age were also independent predictors of adverse outcome. Our findings corroborate the prospective study by Pisters et al. examining 1041 patients with localized soft tissue sarcoma of the extremities, where disease specific survival rates were worse in tumors of higher grade, larger size, positive surgical margins, and specific histological subtypes. [10, 13] A specific histological subtype analysis was beyond the scope of our study. A study by Singer et al. retrospectively analyzed a prospective database of 182 patients with extremity sarcomas and similarly found that grade, size, histological subtype and age were prognostic of outcome. [14] The findings of both the Pisters study and the Singer study have been borne out in other studies as well. [15–20] Altogether, our findings are important affirmations of the current literature and add credence to our model.

Two studies have previously investigated the role of race on STS-E outcomes using large population databases. These studies had conflicting results. A study by Martinez et al. retrospectively examined 6406 adult patients from 1988 to 2003 in the SEER database; in a multivariate analysis, they found that black patients had worse outcomes than white patients, while Asian patients had superior outcomes overall. [8] In contrast, Alamanda et al. retrospectively examined 7601 patients from 2004 to 2009 in the SEER database; they found that, while there are discrepancies in both patient and tumor characteristics between races, black race alone is not an independent driver of outcome. [7] These discrepancies may be explained by a few factors. First, these studies examined different time periods. Second, both studies utilized the SEER database, which has limited information regarding treatment characteristics and only captures about 28% of the population. In contrast, the NCDB captures more than 70% of new cancer diagnoses from nearly 1500 accredited CoC institutions and contains far more detail regarding treatment characteristics. The most striking aspect of our study, which had more patients than both studies combined, was that black race was identified as an independent predictor of adverse outcome, demonstrating a 25% increased rate of mortality independent of tumor or treatment specific factors as compared to white patients.

We also found surprising differences in both tumor and treatment characteristics between races. Black patients tended to have larger tumors, though interestingly, white patients had higher grade tumors than black or Asian patients. Black patients had higher rates of amputation; however, 10% of black patients had no surgery, compared to 6.37% and 7.45% in white and Asian patients. There were no statistically significant differences in rates of margin positive surgery. The studies by Martinez and Alamanda are consistent with our findings, showing that black patients had a trend towards higher rates of amputation or no surgery at all. With regards to adjunct therapies, our study identified no difference between rates of radiation therapy or chemotherapy treatments among patients of different races, nor was there a difference in time to treatment for these treatment modalities. Unlike our study, the study by Alamanda et al. suggested that African Americans had lower rates of RT usage overall, which held true in their multivariate analysis. Overall, these findings suggest that there are racial differences with regards to tumor characteristics at the time of diagnosis and treatment decisions. The average larger size, but lower histologic grade of the tumors at the time of diagnosis may represent a disparity in time to diagnosis. It is unclear however, whether differences in treatment decisions is related to patient preference, surgeon recommendation, or extent of disease. Perhaps the most notable finding in this study is the confirmation that survival outcomes did not improve over the study period for black patients.

While we felt that our study represented a suitable model for the population as a whole, it is not without limitations. Perhaps most important, this is a retrospective cohort analysis of a large population database. While contributing components are accredited CoC institutions, the database is only as powerful as its contributing components; while extensive efforts are made to ensure the sanctity and completeness of the data, there is always a chance that information was entered erroneously or incompletely. What is more, this database does not include information about local recurrence or recurrence free survival, which is an important point of consideration when discussing sarcoma outcomes. Overall survival data was not available for patients diagnosed after 2007. It is possible that in the past 10 years, outcomes have changed for patients of different races. Additionally, it is important to acknowledge that overall survival is not necessarily the same as disease-specific survival. However studies looking at overall cancer survival suggest that little improvement has been made regarding racial disparities over the last 20–25 years. [11] These limitations, and those of the studies preceding ours, point to the need for a well-designed prospective study looking for race specific differences in outcome and the specific reasons underlying these differences.

This large database review alarmingly reveals statistically significant inferior outcomes of black patients with STS-E. These include larger tumors, poorer socioeconomic status with lower rates of insurance and poorer education, a greater likelihood of amputation, and decreased survival than white and Asian patients. Future studies are warranted to better understand the etiology of these differences, so that we may attempt to target and narrow these disparities in the future, ensuring adequate access to effective treatment for all patients.

## Abbreviations

CCS: Charlson Comorbidity Score
CoC: Commision on Cancer
ICD-O-3: International Classification of Diseases for Oncology, third edition
NCDB: National Cancer Database
SEER: National Cancer Institute’s Surveillance, Epidemiology and End Results
STS-E: Soft tissue sarcoma of the extremities
